# Esophageal Gastrointestinal Stromal Tumor with Rare Intracranial Metastasis

**DOI:** 10.1155/2020/8842006

**Published:** 2020-10-11

**Authors:** José Carvalho, Margarida Teixeira, Francisco Teixeira Silva, Alexandra Esteves, Carlos Ribeiro, Diana Guerra

**Affiliations:** ^1^Serviço de Medicina Interna, ULSAM—Hospital de Santa Luzia, Viana Do Castelo, Portugal; ^2^Serviço de Anatomia Patológica, Hospital de Braga, Braga, Portugal

## Abstract

*Introduction*. Gastrointestinal stromal tumors (GISTs) are mesenchymal tumors and constitute the largest group of nonepithelial digestive neoplasms. However, they do not represent more than 1% of primary digestive tumors. They commonly metastasize to the liver and peritoneum, but brain metastases are extremely rare. *Clinical Case*. A 76-year-old woman with a diagnosis of esophageal GIST with liver and lung metastases for 13 years, medicated with imatinib, is presented. She was brought to the emergency department after falling and due to changes in behavior and vertigo with 24 hours of evolution. On physical examination, she presented changes in behavior, dysarthria, dysmetria on the right, gait imbalance, and no motor or sensory deficits. On brain computed tomography and posteriorly on magnetic resonance, 2 lesions were observed, left frontal and right cerebellar, compatible with metastatic lesions. After contribution of neurosurgery, histology was obtained that confirmed the lesions were GIST metastases. Imatinib was maintained, and whole brain radiotherapy was performed. After 6 months, she died. *Discussion*. The rarity of GIST brain metastases is noteworthy, and because of that, there is not enough experience to be certain of the best treatment. Our patient lived for 13 years with excellent disease control with imatinib, but the fact that it does not cross the blood-brain barrier makes it not useful in preventing or treating brain lesions. New tyrosine kinase inhibitors that may cross the blood-brain barrier could be the answer to these cases.

## 1. Introduction

Gastrointestinal stromal tumors (GISTs) are mesenchymal tumors and constitute the largest group of nonepithelial digestive neoplasms; however, they do not represent more than 1% of primary digestive tumors [1]. They are more commonly found in the stomach, jejunum, and ileum and rarely arise from the esophagus [2].

GISTs may present with malignant behavior, depending on the mitotic rate, primary site, size, and metastases, in up to 30%. When there are metastases, they commonly are found in the liver and peritoneum. Brain metastases are extremely rare [2].

Imatinib, a tyrosine kinase inhibitor (TKI), used to treat GISTs with outstanding success, does not cross the blood-brain barrier and seems to have a limited role in treating patients with intracranial metastases [3, 4].

We present a case of an esophageal GIST treated with imatinib with success, but that presented later with rare intracranial metastasis.

## 2. Clinical Case

A 76-year-old woman with a diagnosis of esophageal GIST with liver and lung metastases for 13 years, medicated with imatinib 400 mg per day, was brought to the emergency department after falling. She presented changes in behavior, dysarthria, dysmetria of the right limbs, and gait imbalance with 24 hours of evolution. She had no sensory or motor deficits.

**Figure 1:**
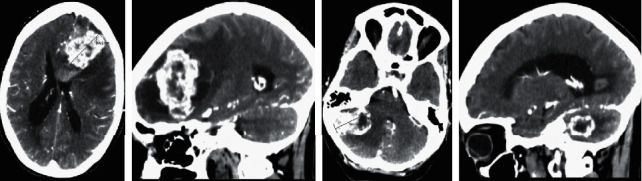
Enhanced CT scan showing 2 lesions with halo of edema, left frontal and right cerebellar. The frontal lesion had an associated bleeding area of 3 cm in diameter.

On brain computed tomography (CT), 2 contrast capturing lesions with halo of edema were observed, left frontal and right cerebellar. The frontal lesion had an associated bleeding area of 3 cm in diameter. The findings were suggestive of metastatic lesions (Figure 1).

Thirteen years earlier, she was started on imatinib with an excellent result, with resolution of lung and liver metastases, maintaining only a stable esophageal thickening over the years.

**Figure 2:**
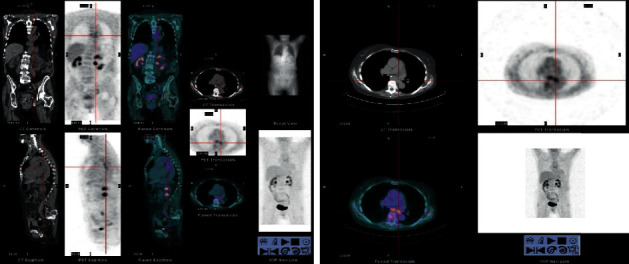
PET-CT showing an already-known metabolically active esophageal lesion, with possible adjacent ganglion metastasis.

**Figure 3:**
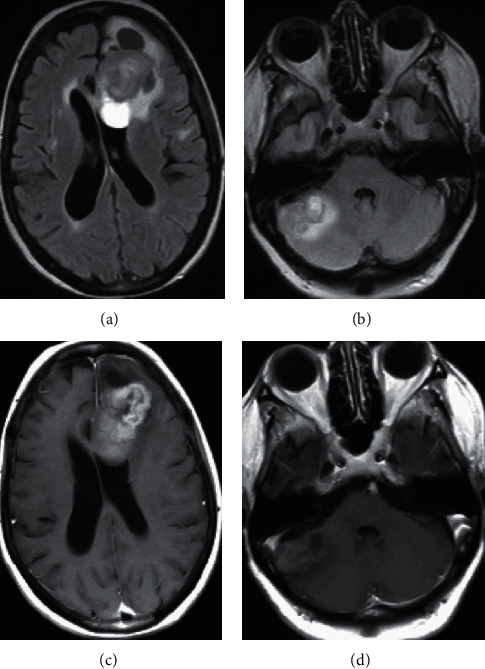
MRI showing the same lesions seen in the CT scan, suggestive of metastases. (a), (b) T2-FLAIR; (c), (d) contrast-enhanced T1-weighted.

As GIST brain metastases are rare and to better characterize the lesions, a positron emission tomography computed tomography (PET-CT) and a magnetic resonance imaging (MRI) of the brain were obtained. PET-CT described an already known metabolically active esophageal lesion, with possible adjacent ganglion metastasis (Figure 2). MRI confirmed the 2 lesions as being compatible with brain metastases (Figure 3).

**Figure 4:**
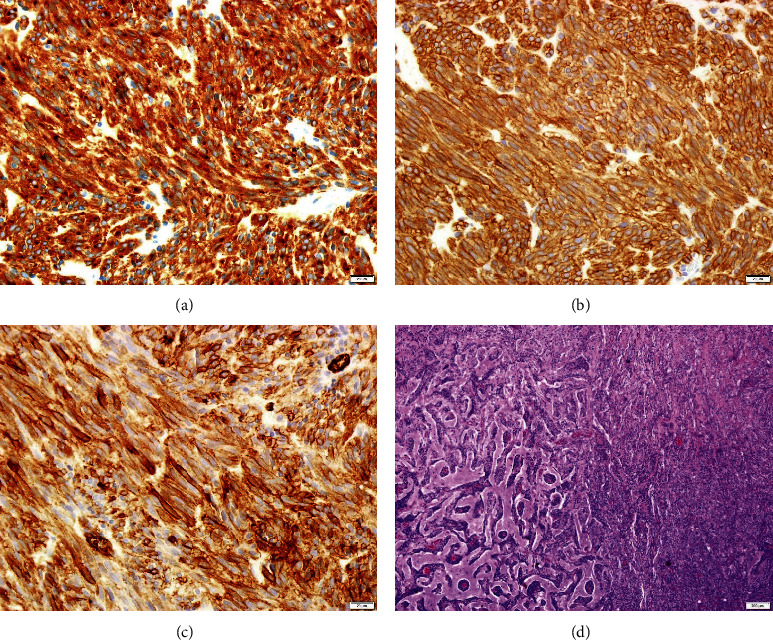
Spindle cell neoplasm with an immunohistochemical study showing positivity for CD117 (a), DOG1 (b), and CD34 (c).

After contribution of neurosurgery, an excisional biopsy of the frontal lesion was performed and histology was obtained, showing a spindle cell neoplasm whose immunohistochemical study showed positivity for CD117, DOG1, and CD34, being compatible with GIST metastasis (Figure 4).

The treatment option was to maintain imatinib and perform whole brain radiotherapy, but without improvement. Her clinical state progressively deteriorated until she died approximately 6 months after the first admission.

## 3. Discussion

The rarity of GIST brain metastases is noteworthy [2, 5, 6]. Only few cases are described in the literature, and because of that, there is lack of experience and evidence to be certain of the best treatment [2]. Our patient lived for 13 years with excellent disease control with imatinib, but the fact that it does not cross the blood-brain barrier makes it not useful in preventing or treating brain lesions.

There is one case [3] that describes reduction of brain lesions with the use of sunitinib, another TKI, that penetrates the blood-brain barrier, but with such few cases, it is difficult to prove its advantage in relation to resection or radiotherapy. Nevertheless, TKI that may cross the blood-brain barrier could deserve research in the future.

## Data Availability

The data used to support the findings of this study are available from the corresponding author upon request.
